# Microstructure Transformation on Pre-Quenched and Ultrafast-Tempered High-Strength Multiphase Steels

**DOI:** 10.3390/ma12030396

**Published:** 2019-01-27

**Authors:** Yonggang Zhao, Zijie Xiang, Yuanbiao Tan, Xuanming Ji, Ling Zhang, Fei Zhang, Song Xiang

**Affiliations:** 1 College of Materials and Metallurgy, Guizhou University, Guiyang 550025, China; 18786109880@163.com (Y.Z.); xiangzijie1992@163.com (Z.X.); ybtan1@gzu.edu.cn (Y.T.); xmji@gzu.edu.cn (X.J.); fzhang338@163.com (F.Z.); 2 Key Laboratory for Mechanical Behavior and Microstructure of Materials of Guizhou Province, Guizhou University, Guiyang 550025, China; 3 National and Local Joint Engineering Laboratory for High performance Metal Structure Materials and Advanced Manufacturing Technology, Guizhou University, Guiyang 550025, China; 4 College of Material Science and Engineering, Chongqing University, Chongqing 400044, China

**Keywords:** multiphase steels, tempered martensite and pearlite, symmetrical structure, microhardness softening mechanism

## Abstract

High-strength, multiphase steels consisting of pearlite surrounded by tempered martensite were prepared by pre-quenching and ultrafast tempering heat treatment of high-carbon pearlitic steels (0.81% C). The microstructures were analyzed by scanning electron microscopy, electron backscatter diffraction, and transmission electron microscopy. With an increasing quenching temperature from 120 °C to 190 °C, the quenched martensite variants nucleated via autocatalytic nucleation along the interface. Furthermore, the tempered nodules exhibited a distinct symmetrical structure, and the tempered martensite and pearlitic colonies in the group also showed a symmetrical morphology. In addition, a reasonable model was formulated to explain the transformation process from quenching martensite to the multiphase microstructure. When the quenching temperature was set to 120 °C, followed by ultrafast heating at 200 °C/s to 600 °C and subsequent isothermal treatment for 60 s, the multiphase structure showed highest strength, and the pearlite volume fraction after tempering was the lowest. The microhardness softening mechanism for the tempered structures consisted of two stages. The first stage is related to martensitic sheets undergoing reverse transformation and the nucleation of cementite on dislocations. The second stage involves the transformation of austenite into pearlite and continued carbide coarsening in the martensitic matrix.

## 1. Introduction

The design and development of low-alloy steels with excellent mechanical properties at low cost has been a challenge for structural applications. In view of this challenge, many alloy steels, such as transformation-induced plasticity (TRIP) steel and maraging steel, have been developed [1,2,3,4]. Although these steels possess improved mechanical properties in terms of their strength and plasticity compared with low-alloy steels, they can be used only in certain conditions due to their dependence on costly alloying additives [5]. Hence, the design of effective structural steels with improved strength and ductility has become particularly important. Thus, the research and development of various high-strength multiphase steels by proper heat treatment of low-alloy steels is attractive. In recent years, multiphase structural steels have been extensively investigated [6,7,8,9].

The theory of a “multiphase structure” has been a topic of focus as a technology for improving the strength and ductility of steel. Multiphase steels show remarkably enhanced strength without a significant reduction in plasticity, or show improved plasticity without reduced strength. In general, the design for multiphase steel requires an effective combination of hard and soft phases, such as martensite and bainite, pearlite and ferrite, ferrite and martensite, or austenite and martensite [10,11,12]. The soft phase favors plastic deformation, while the hard phase can improve strength. Strain partitioning between the hard and soft phases can remarkably improve the mechanical properties [6,13,14].

In recent years, many studies on the mechanical properties of multiphase steel have been reported in the literature [15,16,17]. Many investigations have indicated that whether low or high, carbon content generally increases the strength of steel by yielding a quenched martensite phase [18]. Zare et al. [12] investigated the effects of the martensite volume fraction on the tensile properties of a ferrite–pearlite–martensite triple-phase microstructure and reported that the strength increased with an increase in the martensite volume fraction. Elliot et al. [19] showed that martensite is three times more effective as a strengthener than pearlite. However, the two phases both have deleterious effects on uniform and total elongation. Hence, annealing was subsequently used to enhance plastic deformation. Furthermore, Li et al. [7] reported that an increase in the tempering temperature reduced the hardness and the yield and tensile strengths of low-carbon ferrite and martensite dual-phase steel. Additionally, a study performed by Varshney et al. [20] investigated the effects of high-temperature tempering on the tensile properties of low-alloy steel with a ferrite–pearlite–martensite triple-phase microstructure. It is interesting to note that the elongation increased significantly with variation in the tempered martensite content. Meanwhile, under this condition, the tensile strength increased with increasing tempered martensite content. Many studies have focused on low-carbon alloy steel, while few have focused on the heat treatment processing of high-carbon steels due to the complex phases and difficult control associated with such steels.

Thus, in this investigation, a combination of pearlite and tempered martensite phases was obtained by isothermal transformation of high-carbon steel in the austenite region followed by pre-quenching and subsequent ultrafast tempering (PQFT) at different temperatures. Furthermore, the microstructure transformation mechanism is discussed, including rapid heating to a highest temperature within the range of 500–700 °C and subsequent rapid heating and tempering. A theoretical analysis coupled with acquired experimental data was then proposed to explain the evolution of microhardness softening.

## 2. Materials and Methods

**Figure 1:**
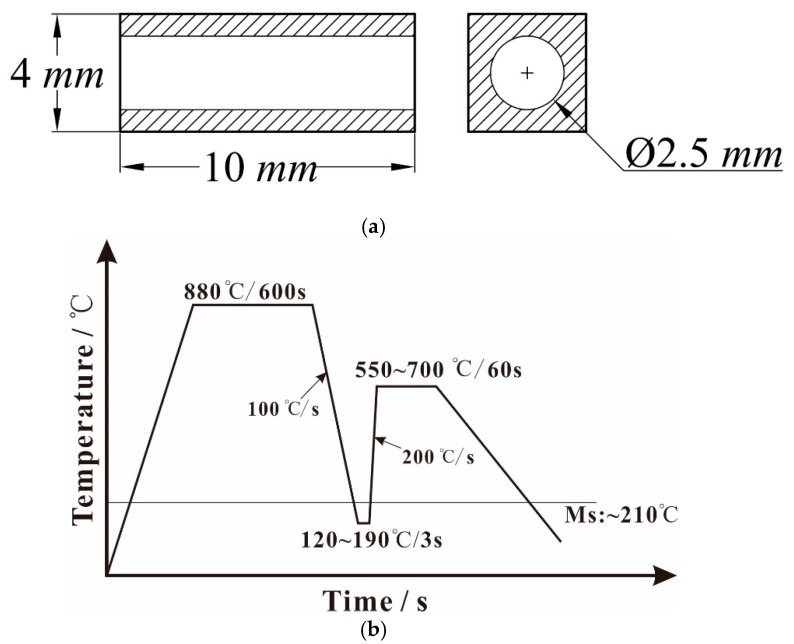
(**a**) Specimen size for the heat treatments on the dilatometer; (**b**) heat treatment process curve of high-carbon steel.

In this study, the experimental material was SWRS82B steel wire with a diameter of 12.5 mm, the chemical composition of which is indicated in Table 1. Heat treatment experiments were performed using a DIL-805A/D dynamic and static dilatometer (BAEHR, Pirmasens, Germany) for precise control of the heating procedure of each phase. The specimen size for the heat treatments and the process curve are shown in Figure 1.

After austenitizing at 880 °C for 600 s, the heat treatment schedules were designed to achieve multiphase microstructures with pearlitic colonies surrounded by tempered martensite microconstituent volume fractions. The specimens were quickly pre-quenched to different temperatures below the *M_s_* point (120, 150, and 190 °C, held for 3 s) at a cooling rate of 100 °C/s. These rapid annealing cycles were characterized by an ultrafast heating rate of 200 °C/s to different temperatures at 50 °C intervals within the range of 550 °C–700 °C, subsequent isothermal treatment for 60 s, and final cooling to room temperature. Microhardness tests were performed under a load of 200 g on a microhardness tester (HV-1000) with the specimens processed by pre-quenching followed by ultrafast tempering under different temperature conditions. The average of five measurements was recorded as the result of each microhardness test.

The samples were then mechanically polished and etched with 3% nitric acid in alcohol. The microstructure morphologies were examined using a ZEISS SUPRA 40 field emission scanning electron microscope (SEM, ZEISS, Oberkochen, Germany). To characterize the microstructure of the samples after the tempering process, TEM analysis was carried out on a Tecnai G2 F20 S-TWIN (FEI, Hillsboro, OR, USA) operated at a voltage of 200 kV. Samples were prepared by twin jet electropolishing in an alcohol solution of 7% HClO_4_ at a temperature of −20 °C and current of 50 mA. Electron backscatter diffraction (EBSD, ZEISS) analysis was performed to study the crystallographic orientation and morphological characteristics before and after tempering; this was carried out using an HKL EBSD detector mounted on an FEI Quanta 650F with Channel 5 software for electron image capture at 20 kV and a probe current of 80 μA with a working distance of 18 mm. The diffraction data were acquired with a step size of 0.12 μm. Tango and Mambo menus were used for data processing to get the IPF maps and PF maps. A noise reduction menu was performed to clean-up the bad point. The standard noise reduction used to remove Zero solutions and isolated points that have been incorrectly indexed and appear as Wild Spikes. The points that have been removed are filled in using copies of neighboring points. In this test, the orientation of each pixel was obtained for a neighboring pixel pair with 3 × 3 and the smoothing angle set as 5°. Specimens for EBSD characterization were electropolished in a solution containing 250 mL distilled water, 125 mL alcohol, 125 mL H_3_PO_4_, 25 mL isopropanol, and 2.5 g carbamide at an electric current density of 450 mA/cm^2^ for 60 s. 

## 3. Results

### 3.1. Microhardness Performance

**Figure 2:**
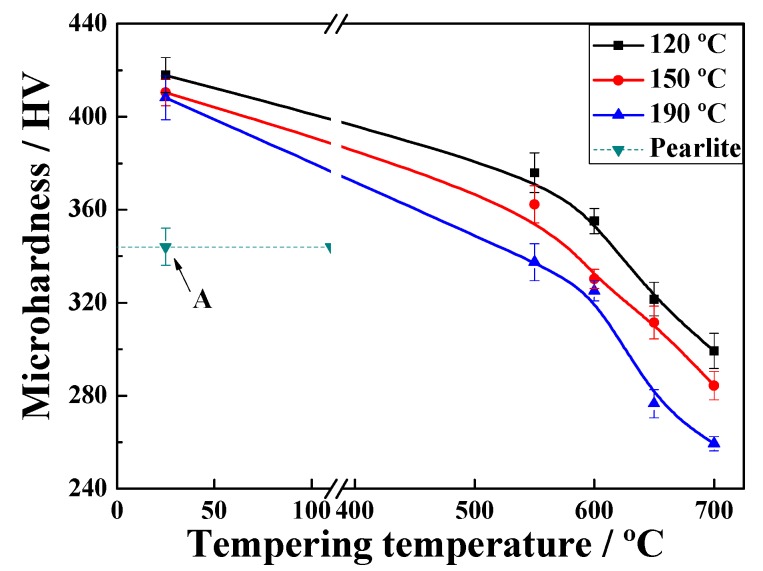
Microhardness curves for samples processed at different quenching temperatures and different tempering temperatures. Black, red, and blue lines indicate the samples quenched at 120 °C, 150 °C and 190 °C, respectively. Point “A” indicates the microhardness of the original pearlitic steel.

Figure 2 shows the microhardness as a function of the annealing temperature. The values at each quenching temperature (QT) clearly show a similar and decreasing tendency with increasing temperature. Furthermore, the QT is low and the rigidity is high at the same tempering temperature. Samples quenched at 120 °C, 150 °C, or 190 °C and tempered at 600 °C were selected for comparison with original pearlitic steel in terms of microhardness. Two other significant reasons for choosing the selected transformation temperature were to avoid the highest bainite start temperature (*B_s_*) and for convenience in controlling the ultimate microstructure.

### 3.2. Microstructure Characterization

**Figure 3:**
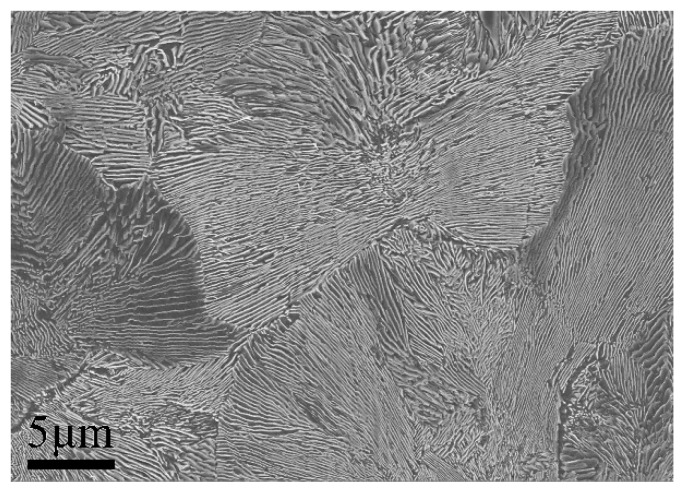
SEM micrographs of initial pearlitic wires.

**Figure 4:**
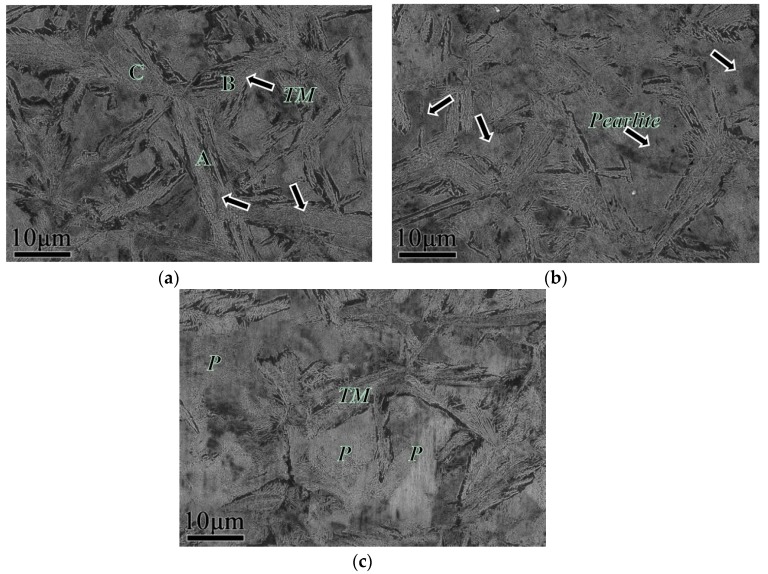
SEM micrographs of the samples quenching at different temperatures, (**a**) 120 °C, (**b**) 150 °C, and (**c**) 190 °C and tempering at 600 °C.

**Figure 5:**
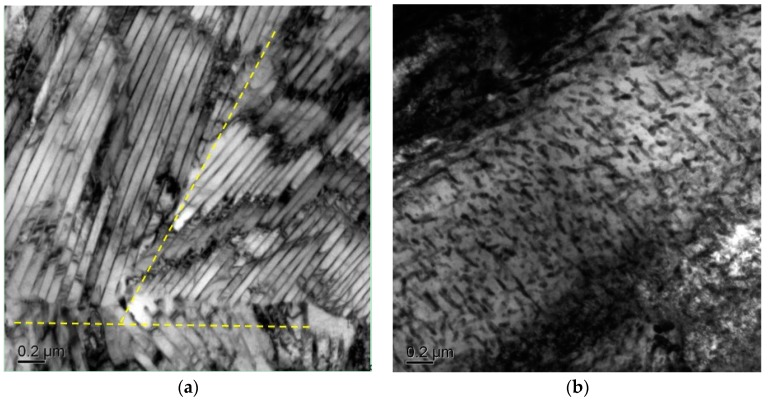
Typical TEM micrographs of pearlitic colonies (**a**) and tempered martensite (**b**) after annealing.

**Figure 6:**
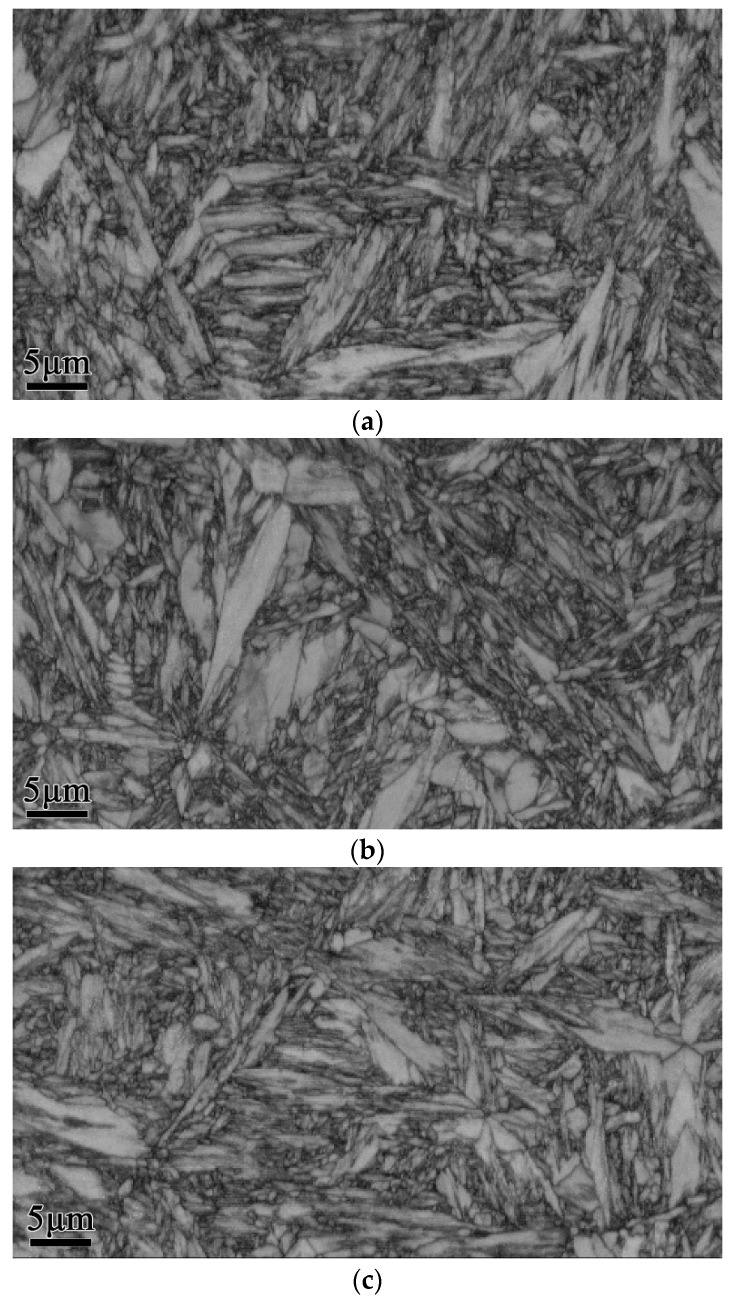

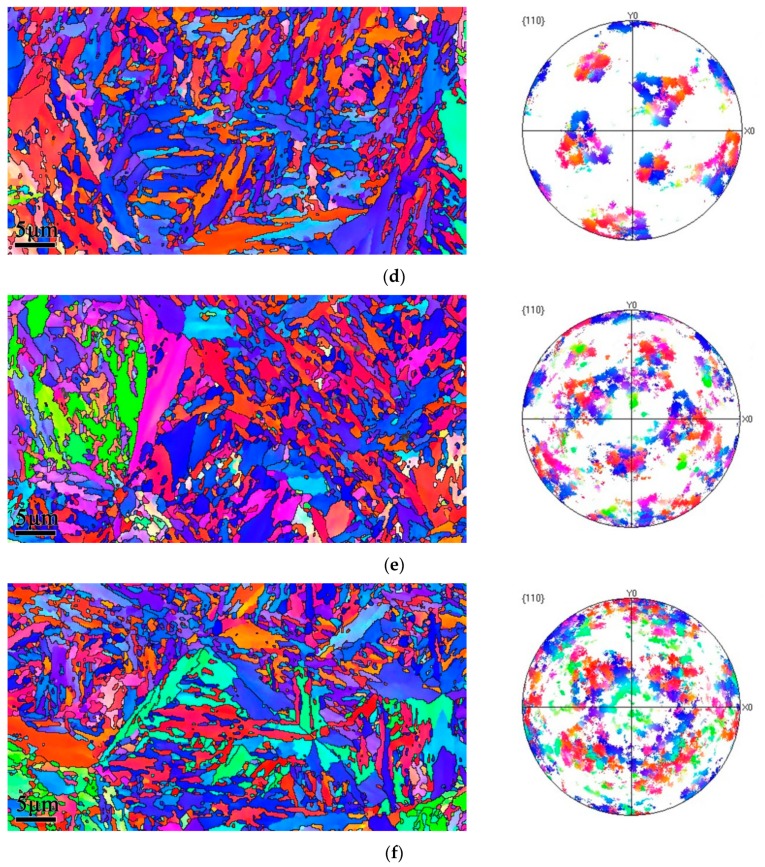

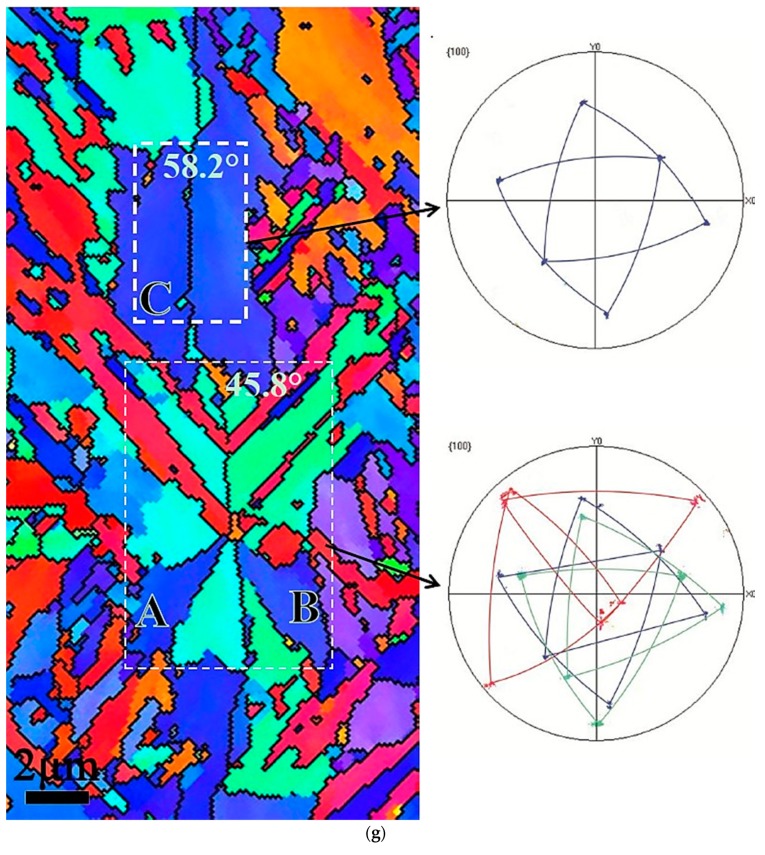
The forescatter detector (FSD) images (**a**–**c**) and the corresponding inverse pole figure maps with the pole figures (PFs) (**d**–**f**) at different quenching temperatures for the martensitic microstructure: (**a**) 120 °C, (**b**) 150 °C and (**c**) 190 °C. (**g**) Local morphology near the boundary and subcollection of PFs of the samples quenched at 190 °C. The black lines indicate the high-angle boundaries when the range of misorientations is 15°–63°.

Figure 3 shows the microstructure of the initial pearlitic wires as observed by SEM. The structure morphology is mainly pearlitic colonies, and exhibits a random orientation. Figure 4 shows the microstructure of three samples at different QTs and tempering at 600 °C for 60 s. The morphologies clearly indicate that a prospective multiphase microstructure was attained, i.e., tempered martensite (TM) surrounding pearlitic colonies. The typical morphologies of TM exhibited dendritic features, as shown in Figure 4a. The pearlitic colony volume clearly decreased with the decline in QT. Furthermore, parts of the tempered martensitic structure maintained a divergent growth pattern at the triple junction that ran through all the prior austenite grains. Figure 5 shows the structure of the cementite morphology of TM, as well as the lamellar microstructure of the ferrite and cementite layers inside the pearlitic colonies, as observed by bright-field TEM. Regardless of the quenching temperature used, the cementite microstructure in the tempered martensite occurred in the form of elliptical particles or short rods, and was dispersed on the TM matrix. The measurement results clearly indicate that the interlamellar spacing (ILS) equaled 98 ± 10 nm and the minor axis of the cementite feature equaled 45 ± 8 nm, where the parameters were measured under edge-on conditions [21,22,23,24]. Moreover, it is interesting to note that the lamellar orientation of the adjacent pearlitic colonies appeared to grow symmetrically at higher quenching temperatures—at 190 °C, for instance. As for the samples quenched at 120 °C, 150 °C, and 190 °C, a clear topography is seen in the forescatter detector (FSD, ZEISS) images (Figure 6a−c ), in which the morphologies exhibit a typical martensite sheet structure.

### 3.3. EBSD Analysis

**Figure 7:**
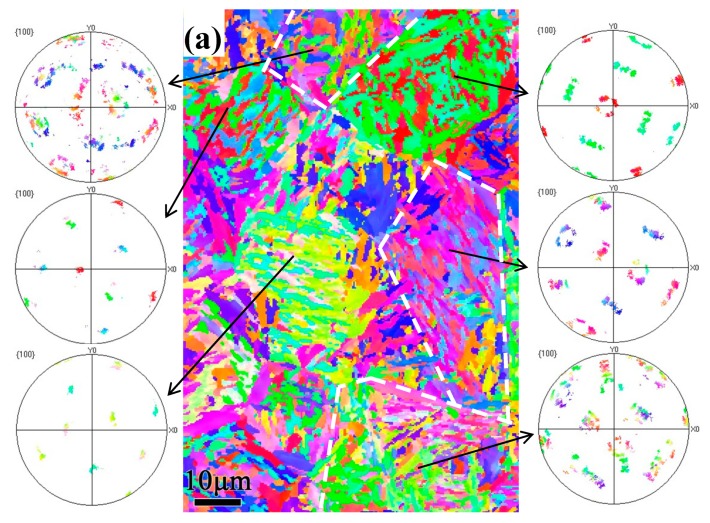

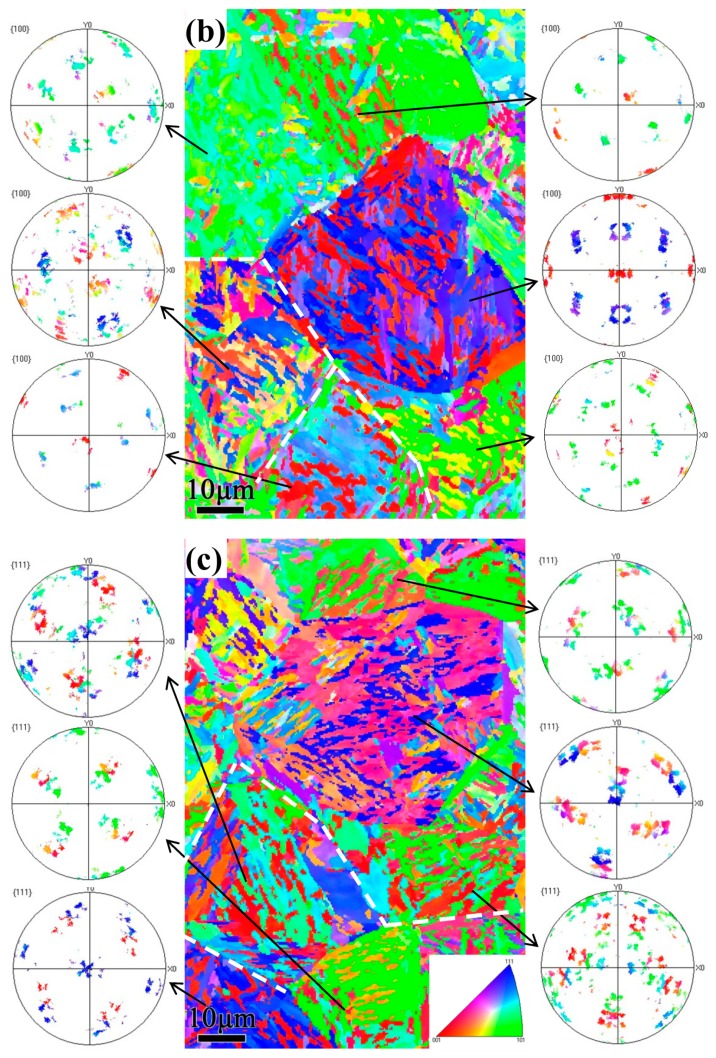
Inverse pole figure maps and the corresponding pole figures of each grain at different quenching temperatures followed by annealing at 600 °C: (**a**) 120 °C, (**b**) 150 °C and (**c**) 190 °C. White lines mainly indicate the selected area of the PF subset. The nodules include the structures of tempered martensite and pearlite colonies.

Electron backscatter diffraction (EBSD) imaging before and after the annealing of specimens was performed to investigate the orientation relationship of the martensitic phase during the transformation and reverse transformation processes. Figure 6d–g shows the inverse pole figure (IPF) maps and the corresponding pole figures (PF) on the right obtained at different QTs. It can be clearly observed from the PF that the microstructure of the quenched samples at 120 °C has a distinct crystallographic orientation relative to that quenched at 190 °C. Thus, for the quenched microstructure, it can be inferred that the crystallographic orientation of the local microdomain became different in orientation as the QT increases, i.e., the microstructure showed isotropic behavior at 190 °C in this microregion. Typical characteristics obtained under this condition can be interpreted from the symmetrical growth of martensite at prior austenite grain boundaries, as shown in Figure 6g. Additionally, the martensite units labeled “A” in Figure 6g appear to have a well-defined crystallographic boundary with units “B”. The orientation relationship between these units was determined to be 45.8°, and the rotation axis/angle was determined to be 58.2° for units “C”. The subcollection of planes {100} for PF showed that the crystallographic orientation of martensitic units was symmetrical. The same analytical method was applied to the samples quenched at different temperatures followed by ultrafast tempering at 600 °C for 60 s. Figure 7 shows IPF maps and the crystallographic relationship of each grain as insets in the PFs, correspondingly. The figure clearly shows that as the QT increases, the symmetry orientation of each nodule gradually becomes more apparent. The typical morphology characteristics are labeled “A”, “B”, and “C” in Figure 4 for TM; these units belong to the same plate group and form a clearly featured coupling to the preceding unit, which may be of the kink or wedge type. This morphology is in stark contrast to the morphology of pearlitic colonies after the tempering observed via TEM, as described above.

## 4. Discussion

### 4.1. Crystallographic Relationship with the Tempering Multiphase Microstructure

It is not possible to reliably use martensitic high-strength alloys in their as-quenched condition without tempering heat treatments. Even when reasonable toughness might be achieved without tempering, there is a tendency for static failure as a result of hydrogen embrittlement occuring during servicing. Thus, most high-strength steels are used in a tempered state. The entire heat treatment process leading from austenite to the multiphase microstructure can be divided into four stages: (1) A complete austenitizing stage, followed by (2) a rapid cooling stage, in which the steel is directly quenched below the *M_s_* point after austenitization. Due to the large degree of supercooling, a large phase transition driving force is produced. Nearly all of the sheared austenite phase is transformed into sheet martensite, the growth of which occurs along the original austenite habit plane. Then, (3) an ultrafast heating stage, during which two changes can be observed. First, carbides in martensite initially nucleate rapidly at the boundaries or in zones of high dislocation density; thus, the density of dislocations and the microhardness decrease dramatically. However, it is not surprising that many reports have indicated that parts of the martensitic sheets will be adversely transformed into austenite [3]. Finally, (4) an isothermal stage, in which the metastable austenite is further transformed into pearlitic colonies and the quenched martensite is decomposed into TM. Two distinct phenomena occur during this work stage. First, the volume of pearlitic colonies increases with QT, and the structure of the TM surrounding the pearlitic colonies is maintained. Second, it is interesting to note that regardless of whether a phase is quenched martensite, ultimately annealed pearlite, or TM in a nodule unit, the crystallographic orientation remains symmetric with increasing quenching temperature, as described above.

The reason for this is that martensite sheets grow along the austenitic habit plan during the shear transformation process, along <259>_r_ at low temperature and <225>_r_ at high QT [25]. Among the martensitic sheets, the first will penetrate the integral austenite grains, and the subsequent martensitic sheet structure will gradually decrease. If the quenching temperature is low, the phase transformation driving force is large, strengthening the shearing ability of martensite sheets along the habit plan and making the crystallographic orientation of the microregion more distinct, as shown in Figure 6. Notably, Albin et al. [26] analyzed the formation of plate martensite in high-carbon, low-alloy steels. It is worth noting that in addition to the formation of a [112]_M_ twin crystal structure, a small amount of [101]_M_ twin crystal formation occurs inside the martensite sheets at low quenching temperature. Thus, due to the higher interfacial energy and pinning effect of the [101]_M_ twin crystal, the reverse transformation behavior is difficult to achieve, and the phase ultimately transforms into TM. The secondary martensite structures, with small layers, a disordered orientation, and a low interface energy, are more prone to reverse transformation and eventually form a pearlite structure. Another reasonable explanation for the abovementioned transformation behavior is that the martensitic sheets travel along low-index crystal planes, e.g., along [225]_r_, at high quenching temperature. Usually, only a [112]_M_ twin orientation occurs inside martensitic sheets. Similarly, researchers have indicated that the dislocation density and the quenching temperature are inversely related [27,28]. Hence, a low dislocation density and low surface energy allow for easier reverse transformation of the microstructure, and the volume of pearlitic colonies is high.

**Figure 8:**
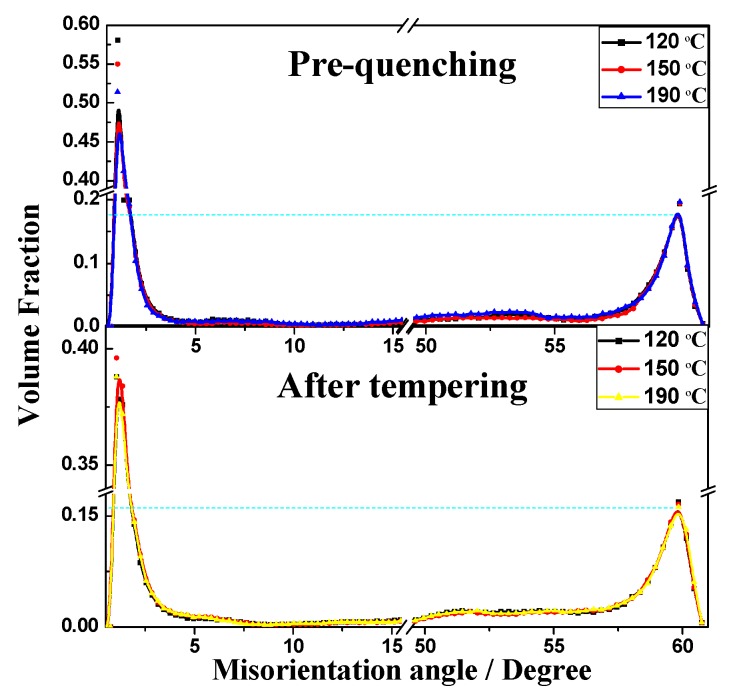
The misorientation angles before and after the tempering process.

The symmetrical microstructure inside a nodule can be characterized by EBSD analysis. Samples pre-quenched at 120 °C, 150 °C or 190 °C and submitted to ultrafast heating (600 °C, 60s) were tested. In contrast to the statistical analysis results pertaining to the misorientation angle before and after tempering, the crystallographic orientation did not change significantly, and only the number of small angles decreased, as shown in Figure 8. It was verified that the effect of tempering only altered the dislocation density, whereas the tempered misorientation structure did not change significantly. Thus, it can be inferred that although the martensite reversibly transformed into austenite, the parallel dislocation channels formed by shearing deformation or residual twins were preserved in the austenite matrix. Therefore, during the diffusion-type growth transformation from austenite to pearlite, carbon atoms more easily migrated and formed cementite lamellae along the defects inside the austenite phase. Ultimately, a symmetrical pearlitic structure formed.

### 4.2. Evolution Model of the Multiphase Microstructure

**Figure 9:**
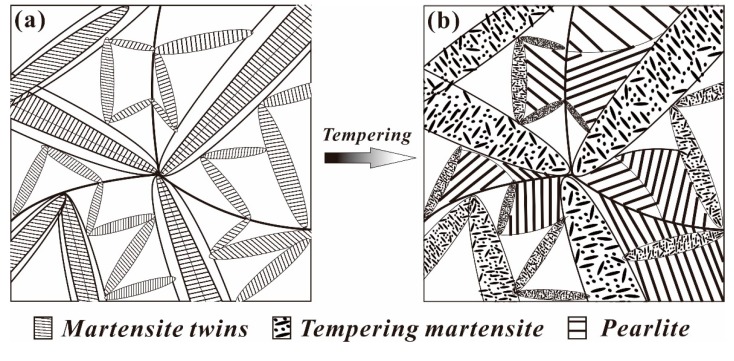
Schematic of the microstructure transformation of quenched martensite into tempered martensite: (**a**) the martensitic structure after quenching and (**b**) the multiphase structure after tempering.

Many studies have reported that new martensite variants are often nucleated via autocatalytic nucleation [20,29]. Furthermore, autocatalysis will generate well-defined kink-type crystallographic boundaries and form wedge-type secondary martensite variants based on primary martensite variants. Both these orientation relationships and their nonrandom nature have previously been investigated and discussed by Okamoto et al. and Stormvinter et al. [26,28]. Similarly, in this study, the abovementioned phenomenon was also observed at different QTs and tempering at 600 °C for 60 s by SEM, as shown in Figure 4. Interestingly, an obvious homologous orientation in microdomains, which is closely related to the orientation of martensite variants during the transformation process, could be observed in samples quenched at 120 °C. Due to the large degree of subcooling, the formation of martensitic variants with the same orientation was possible. A schematic of the evolution of quenched martensite to TM in this type of high-carbon, low-alloy steel is presented in Figure 9, where Figure 9a presents the martensitic structure after quenching, and Figure 9b presents the multiphase structure after tempering. Commonly, the martensite variant of the midrib type without reverse transformation is transformed into tempered martensite, the final morphology is lenticular, and the martensite undergoing reverse transformation forms a pearlite structure. Sometimes, the pearlitic colonies are separated by a structure of banded TM.

### 4.3. Softening Mechanism

**Figure 10:**
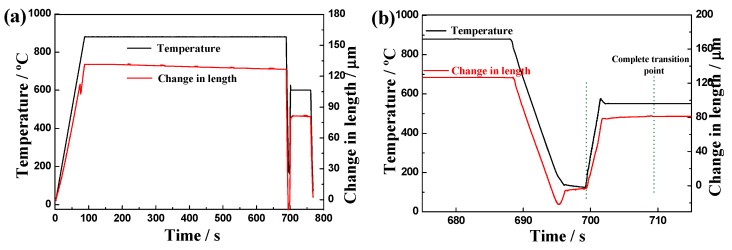
(**a**) Real time–temperature–expansion curve of the heating process for samples pre-quenched at 120 °C and tempered at 600 °C; (**b**) local curves for “temperature” and “change in length” transformation.

**Figure 11:**
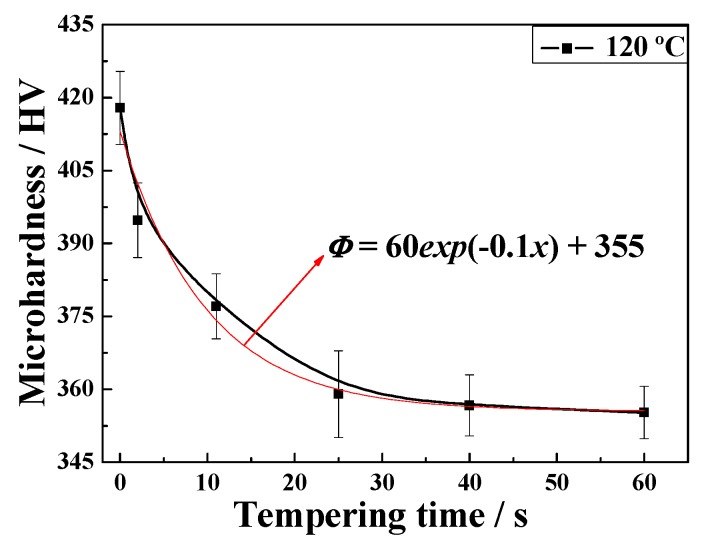
Microhardness of samples quenched at 120 °C and tempered for different lengths of time.

The time-temperature-expansion curve of samples heat treated at a QT of 120 °C and a tempering temperature of 600 °C is presented in Figure 10. The black line represents the actual heat treatment temperature curve, and the red line represents the length change curve of the sample during heating and cooling. The length change curve is closely related to the microstructure transformation process. After annealing for 600 s, the expansion curve was nearly flat, demonstrating that the sample had completely changed from the structure of pearlite to austenite. In the subsequent rapid heating process, the length of expansion was smaller than that in the case of complete austenitization, which indicates that only part of the martensite structure was reverse-transformed into an austenite structure. Thus, as shown in Figure 10b, the time required for complete pearlitic isothermal transformation was approximately 10 s. Furthermore, the microhardness of samples tempered for different durations was tested under these conditions, as shown in Figure 11; the microhardness decreased according to a negative exponent [30] and satisfied the formula:

(1) Φ=60exp(−0.1x)+355

where Φ is the microhardness value along the fitted curve and x is the tempering time.

Researchers have reported that there are two stages of microstructure transformation in the fourth step, i.e., ultrafast heating and annealing [19,29,31]. The first stage is rapid heating. Xing et al. [32] investigated the effect of refined precipitation on the high-temperature rapid tempering process of SS400 steel. The results reflected that cementite tended to be refined and dispersed if the heating rate exceeded 3 °C/s. Contributions to ultrafast heating during the tempering process, determined using a thermomechanical simulation tester at a heating rate of 200 °C/s, could cause the temperature to reach higher levels so rapidly that there was insufficient time for cementite precipitates to grow along the boundaries [30]. This process was accompanied by rapid carbide nucleation on dislocations in less than one second [19]. Furthermore, parts of martensitic sheets reversibly transformed into austenite. Hence, the softening mechanism in the first stage was related to the microstructure transformation as well as carbide nucleation. As expected, structural transformation occurs following the subsequent isothermal tempering process, i.e., when austenite transforms into pearlite, and carbide coarsening continues to occur in the martensitic matrix, which is the second stage of softening. Therefore, the microhardness in the early stage decreases greatly at a tempering time of less than 11 s. Thus, microstructure transformation is the dominant factor in this process. Elliot et al. [19] proposed that carbide-coarsening-induced softening behavior decreased linearly with tempering time within 10 s. The slow microhardness reduction observed later was caused by the coarsening of cementite.

## 5. Conclusions

In this study, high-strength steels containing multiple phases consisting of pearlite surrounded by tempered martensite were formed via the PQFT heat treatment of high-carbon pearlitic steels. The evolution of microstructure transformation was investigated, and the following results were obtained:

(1) The values at each quenching temperature clearly show a similar and decreasing tendency with increasing temperature. When the quenching temperature was set to 120 °C and isothermal treatment at 600 °C for 60 s, the multiphase structure showed highest strength, and the pearlite volume fraction after tempering was the lowest.
(2) When the quenching temperature is higher, e.g., at 190 °C, the quenched martensite sheet nucleated via autocatalytic nucleation along the interface and showed an obvious symmetrical morphology.
(3) After heat treatment process, the microstructure inside a nodule containing the pearlitic colonies and TM, the crystallographic orientation remains symmetric with increasing quenching temperature.
(4) The microhardness of the tempered microstructure decreases with increasing quenching temperature and tempering temperature. In addition, the microhardness decreases according to a negative exponent for tempering time within 60 s.

## Figures and Tables

**Table 1:** Chemical composition of the cold drawn pearlitic steel wires used in this study (wt %).

C	Si	Mn	P	Cr	Fe
0.810	0.180	0.840	0.014	0.272	Bal.
